# On the Action of Salicylates in Certain Rheumatic Diseases

**Published:** 1891-08-22

**Authors:** 


### ON THE ACTION OF SALICYLATES IN CERTAIN RHEUMATIC DISEASES.

Thanks to the labours of Cheadle and other investigators, it is now recognised that we can no longer speak of endocarditis or pericarditis as a complication of rheumatic arthritis, but that these three diseases, as well as certain others, are all results of the action of the rheumatic virus—that this rheumatic virus, whatever be its nature, may produce, in addition to the above, tonsillitis, erythema nodosum, rheumatic nodules, chorea, and pleurisy—so that if, *e.g.*, any two of these occur simultaneously or in succession, we cannot say the one is primary and the other dependent on the first. They are all, so to speak, on a level.

Of these diseases some, *e.g.*, rheumatic nodules, are produced solely by the rheumatic poison; others, *e.g.*, pericarditis in children, are rarely due to any other cause, whilst others again, *e.g.*, chorea, may own a quite different origin.

We have several means of finding out whether any one of the above is rheumatic in nature—there is the family history, the personal history, and the effect of treatment. The family history is of great value, for it has been shown that a child suffering from a rheumatic disease is four times as likely to have blood relations, who have had some rheumatic manifestation, as is a child who is not so suffering—there is an hereditary tendency. The important point, however, is whether any other disease, which may result from the action of the rheumatic poison, is or has been present—thus, if a child who is suffering from serous arthritis is found to have an endocardial murmur, the two are pretty certain to be rheumatic in nature; similarly, as Cheadle says, an endocardial murmur generally picks out the rheumatic cases of chorea.

These two facts being pretty certain, it is somewhat surprising to find it laid down that of all these rheumatic manifestations only tonsillitis and arthritis are rapidly relieved by salicylates. A short experience, however, suffices to show that this statement, if not absolutely, is yet approximately true. But one is tempted to ask whether salicylates will not beneficially influence some other rheumatic manifestations even though there is no such rapid cure as in the case of tonsillitis and arthritis. And it would appear to us, from an experience of some thirty cases of various combinations of these rheumatic manifestations, that rheumatic chorea and rheumatic enythema are benefitted by the administration of salicylates—that [on comparing the cases in which salicylates have been, with those in which they have not been given] recovery has been more rapid, and that there has been a diminution in the number of other rheumatic manifestations.

It is as yet an unsolved problem why salicylates do not cure some diseases, *e.g.*, rheumatic endocarditis, whereas they will cure others, for example, rheumatic arthritis. We must wait until we know more accurately the nature of this rheumatic poison. The researches of Latham and Haig go some way towards this, but much more investigation is necessary.

